# Geographic information systems-based expert system modelling for shoreline sensitivity to oil spill disaster in Rivers State, Nigeria

**DOI:** 10.4102/jamba.v9i1.429

**Published:** 2017-07-28

**Authors:** Olanrewaju Lawal, Charles U. Oyegun

**Affiliations:** 1 Department of Geography and Environmental Management, University of Port Harcourt, Nigeria

## Abstract

In the absence of adequate and appropriate actions, hazards often result in disaster. Oil spills across any environment are very hazardous; thus, oil spill contingency planning is pertinent, supported by Environmental Sensitivity Index (ESI) mapping. However, a significant data gap exists across many low- and middle-income countries in aspect of environmental monitoring. This study developed a geographic information system (GIS)-based expert system (ES) for shoreline sensitivity to oiling. It focused on the biophysical attributes of the shoreline with Rivers State as a case study. Data on elevation, soil, relative wave exposure and satellite imageries were collated and used for the development of ES decision rules within GIS. Results show that about 70% of the shoreline are lined with swamp forest/mangroves/nympa palm, and 97% have silt and clay as dominant sediment type. From the ES, six ranks were identified; 61% of the shoreline has a rank of 9 and 19% has a rank of 3 for shoreline sensitivity. A total of 568 km out of the 728 km shoreline is highly sensitive (ranks 7–10). There is a clear indication that the study area is a complex mixture of sensitive environments to oil spill. GIS-based ES with classification rules for shoreline sensitivity represents a rapid and flexible framework for automatic ranking of shoreline sensitivity to oiling. It is expected that this approach would kick-start sensitivity index mapping which is comprehensive and openly available to support disaster risk management around the oil producing regions of the country.

## Introduction

Hazards are usually present across any landscape. However, when adequate and appropriate actions are not taken, such hazards can result in disaster. Therefore, it is apparent that hazards are inevitable; it is the human response and impacts that often dictate whether such hazards become disasters. Oil spills across any environment are very hazardous in relation to their social, environmental and economic impacts. It is with this understanding that oil spill contingency planning is very important in the marine, estuarine, lacustrine and riverine areas of the country. This is especially of significance for those areas where oil exploration and exploitation activities are taking place – the vulnerable regions. After any spill, the situation can quickly deteriorate with huge levels of impact when appropriate and prompt actions are not taken. However, in order to define priorities, initiate actions and deploy resources for oil spill response, there is a clear and definite need for locations of sensitive habitats to be known.

The Exxon Valdez shipwreck 1989 (Alaska) was a wake-up call, which led to the development of the International Convention on Oil Pollution Preparedness, Response and Cooperation in 1990 (OPRC 90) by the International Marine Organization (IMO). The convention came into force in May 1995 and Nigeria is a signatory to the convention. This is aimed at fostering cooperation among countries towards international efforts in emergency preparedness in response to accidents with oil spills. It also stipulated that all ships and offshore facilities in the sector must have emergency plans or similar arrangements coordinated with national systems for response and effective management of oil pollution incidents.

Environmental Sensitivity Index (ESI) mapping is an important tool in oil spill planning. It provides a support for the development of response strategies for oil spills, identification of elements at risk and helping to define and prioritise areas for protection and remediation. ESI has three components: shoreline sensitivity ranking, biological and human resources distribution. Shoreline sensitivity is very important in defining ease of clean-up as well as potential for natural clean-up. With the importance of this tool, it is therefore pertinent that methods and techniques are developed to take into cognisance the challenges of data collection in the middle- and low-income countries. A significant data gap exists across many of these countries in the aspect of environmental monitoring (Gutierrez 2010) and when these data sets are available they are often sparse. It is to this end that this study seeks to develop an expert system (ES) to rank the sensitivity of the shoreline of Rivers State (a major oil producing state in Nigeria) to oil spill using remote sensing and geographic information systems (GIS). This is intended to provide a rapid assessment framework which could lead to the development of ESI and allow for the updating and improvement of oil spill contingency planning in the region and the country, while also supporting effective disaster risk management across vulnerable regions of the country.

Oil spill could be as a result of an accidental release of oil from platforms, tankers, wells, rigs, and so forth into either the marine or terrestrial environments. Over the years there have been numerous examples of significant oil spill in Nigeria; the work of Nwilo and Badejo (2006) and a look at the Oil Spill Monitor (https://oilspillmonitor.ng/) give a clear indication that the occurrence of such disasters is still prominent across the oil producing regions of the country. It is very clear that even with the best laid plans, the management of disasters requires an iterative and continuous evolution of techniques, actions and measures. It is a clear understanding that the impacts of oil spills are often worse because of the lack of adequate data and information to guide actions and deployment of resources for preparedness and response.

Oil spill contingency planning and response are ultimately aimed at protecting human life, reducing environmental impact of the spill and supporting remediation actions (Jensen, Halls & Michel 1998). These goals cannot be effectively achieved if the position of sensitive environments and elements at risk are unknown, not collated and improperly documented. The development of such planning culminates into the production of an ESI map. This map, while supporting the oil spill contingency planning, is also relevant prior to spills in the identification of vulnerable locations, deployment and establishment of protection priorities as well as identification of clean-up strategies (National Oceanic and Atmospheric Administration [NOAA] 2016). ESI mapping has been carried out across many regions of the world in an effort to produce an evidence-based response system for oil spill risk management, especially across many high income oil producing countries. In the United States, the NOAA is responsible for mapping the coastal regions (as well as rivers, lakes and estuaries) of the country. ESI maps are increasingly being developed across many countries and their use is becoming very important in many oil producing countries (Pincinato, Riedel & Milanelli 2009). The NOAA guideline provides a very clear framework for ESI mapping based on physical, biological and human resources present in the environment of interest. According to Pincinato et al. (2009), the NOAA framework formed the backbone of the development of the manual by the Regional Association of Oil, Gas and Biofuels Sector Companies in Latin America and the Caribbean (ARPEL) and International Petroleum Industry Environmental Conservation Association (IPIECA) for ESI mapping. The importance of this tool cannot be overemphasised in the management of oil spills (human-induced hazards).

Even though there are established frameworks for mapping environmental sensitivity to oil spills, data and the condition of the environment often affect how this tool is developed and utilised. The development in the area of GIS and remote sensing technologies as well as increasing access to medium resolution data at global level offers a good opportunity in many parts of the world where data are a major challenge. GIS has unique advantages over the conventional paper-based ESI maps. It allows for reduced production cost because it can be deployed electronically, ensure easy distribution as well as support the ease of updating the map when new information becomes available. Furthermore, using GIS in the development of ESI allows for building of databases which can be queried and provides a framework for extensive spatial analysis.

In recent times, various studies have adopted different methods in the development of ESI map. For example, working on the Mediterranean coast of Israel, Adler and Inbar (2007) used GIS for the development of sensitivity mapping, the relative sensitivity of shoreline types and prioritisation scheme for the shoreline types and coastal resources. Pincinato et al. (2009) implemented the standards set by the Brazilian Federal Environment Organ for the littoral regions of Brazil where oil transportation is intense. The study systematically ranked the littoral regions based on winter and summer condition of the habitat, using a knowledge-based ES. Wieczorek, Dias-Brito and Milanelli (2007) in their study developed a littoral sensitivity index for the Cardoso Island State Park (Sao Paulo State, Brazil). They identified differences in seasonal sensitivities and concluded that estuarine mangroves were the most sensitive of the coastal habitats in the study area. Using a multi-criteria evaluation, Vafai, Hadipour and Hadipour (2013) combined fuzzy set theory, hierarchical structure analysis and the analytical hierarchy process (AHP) within GIS in the development of environmental sensitivity of the coastal area of Mazandaran Province (Northern Iran). The comparison of their results with the actual shoreline indicated that the method adopted (Fuzzy AHP) predicted sensitivity optimally. They attributed this to the combination of fuzzy set theory and the AHP.

In the case of Nigeria, the work of Adeofun and Oyedepo (2011) examined the sensitivity of Atlas Cove in Lagos. The work identified nine classes of sensitivity along the study area based on the NOAA and the Nigerian Oil Producing Trade Section standard. Gundlach, Hayes and Getter (1981) also produced a sensitivity map for oil spills along the Nigeria Coast. Furthermore, the National Oil Detection and Response Agency (NOSDRA) was reported in 2010 to have begun the development of ESI for management of oil spills along the Nigerian Coastline (Mohammed 2010). Fabiyi (2002) also proposed and presented a method of ESI mapping in a selected area of Rivers State; however, this method only considered soil and land use data, excluding other relevant data on shoreline types, biological resources and human resources. From the foregoing, it is quite clear that there is limited work on ESI across the Nigeria marine, estuarine, lacustrine and riverine environments. This could be attributed to data access and availability as well as the enormity of the resources required to complete such tasks. However, there is a need for such information to be developed and accessible to communities and other stakeholders to support awareness and advocacy in the area of environmental justice. To this end, this study presented an approach which could leverage available information and models in identifying the shoreline sensitivity aspect of the ESI mapping exercise.

By simulating the response decision of an expert for a given situation at a given location, a computer program can be developed based on previous knowledge collected in the field or from standard documents (manuals, guideline, etc.). Such understanding or expert knowledge can be provided to the computer system to develop rankings, which can be used to designate the sensitivity index for the different segments of the shoreline. ES represents a branch of applied artificial intelligence whereby the vast body of knowledge for a specific task is transferred from humans to the computer (Liao 2005). ES deployed with the aid of geographic databases has witnessed widespread applications across different areas of research, including mapping of forest soils (Skidmore et al. 1996), predicting Matsutake mushroom habitat in Yunnan, South west China (Yang et al. 2006) and monitoring of salinity (Giordano & Liersch 2012; Metternicht 2001). Furthermore, Lukasheh, Droste and Warith (2001) presented a review of the application of these systems in landfill design and management, showcasing extensive examples. Other examples include mapping of invasive species in Zimbabwe (Masocha & Skidmore 2011), disaster assessment (Kou, Ergu & Shi 2014), siting of retail stores (Sadler 2016), landslide susceptibility (Bui et al. 2012), mineral exploration – potential modelling (Porwal & Carranza 2015), et cetera.

## Materials and methods

### Study area

**FIGURE 1:**
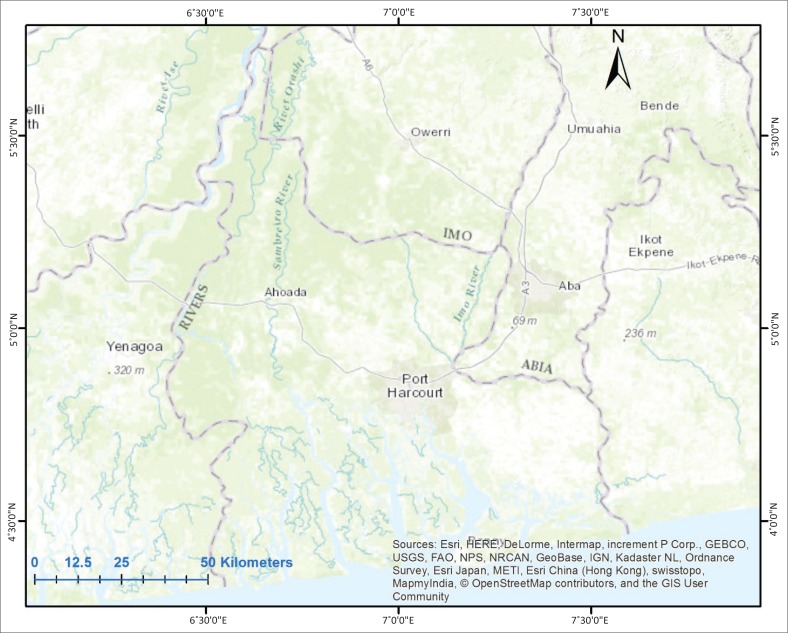
Rivers State topography and drainage networks.

Rivers State (Figure 1) is located within the Niger Delta region of Nigeria with three distinct ecological zones – mangrove forest and coastal vegetation, fresh water swamps and lowland rainforest (Niger Delta Development Commission 2006). The State is intersected by a network of rivers, streams and creeks, with its coastline forming part of the West African Coastline.

Geologically, it is situated within the coastal plain belonging to the Niger Delta sedimentary formation (Short & Stauble 1967). Essentially, the area is on an extensive fluvial alluvium. Slope of the study area ranges between an average of 3 and 5 degrees in an NW-SE direction. Its poor drainage could be attributed to the low relief and gentle slopes found across the entire study area. Details of the region’s land and its people are extensively addressed by the Niger Delta Development Commission (2006). The pattern of settlement across the region is a result of dry land availability; therefore, large cities such as Port Harcourt are usually found on the dry islands in the mangrove swamp in the hinterland of the Delta. Agriculture and industries as well as fishing and subsistence farming are the main source of livelihood. Industries such as food manufacturing, oil servicing, oil and gas, construction and marine industries are also prominent.

The coastline is dominated by the littoral rainforest interspersed by evergreen rainforest, while a small section is covered by swamp forest; however, as one moves inland the landscape is dominated by swamp forest interspersed by mangrove forest (Sayre et al. 2013) and it is dominated by the species *Rhizophora racemosa* (Nwilo & Badejo 2006).

The climate of the study area is Tropical Monsoonal (Rubel & Kottek 2010), which is characterised by a short dry season and a pronounced wet season, which starts around March and lasts till October with a break around August. The temperature remains relatively constant throughout the year and ranges between a maximum of 28 °C and 33 °C to a minimum of 21 °C and 23 °C. The coastline experiences an average wind velocity ranging between 2 m/s and 5 m/s and this speed can increase to about 10 m/s during heavy rainfall and thunderstorms (Nwilo & Badejo 2006).

The State is a major oil producing region of the Niger Delta, boasting extensive reserves of crude oil and natural gas. It is currently the second largest producer after Akwa Ibom State (Oil Revenue Tracking Initiative 2013) and it currently hosts two oil refining facilities, the liquefied natural gas project (Bonny) and a host of other petrochemical related industries.

### Data

Data sets relevant for the categorisation of the shoreline across the study area were collated, and such data leveraged the advances in remote sensing. In order to categorise the shoreline, data on shore types, slope, wave exposure and sediment types were collated. The elevation data were extracted from the Shuttle Radar Topographic Mission (SRTM) 1 Arc second resolution data set from the U.S. Geological Survey. These were used to compute the slope of the region. The data were extracted in the Georeferenced Tagged Image File Format (GeoTIFF), the horizontal datum is the World Geodetic System 1984 (WGS84 – Geographic) while the vertical datum is the Earth Gravitational Model 1996 (EGM 96) ellipsoid and the vertical unit is meters (USGS 2016).

Shore types were manually digitised and identified from a mosaic of recent Google Earth imageries (especially that of 02 November 2016). Visually identifiable segments of the shoreline up to 40 km inland were classified based on visually identifiable features of the shoreline. The work of Oyegun (1993) was also examined to serve as a baseline for comparison of shoreline characteristics along the coastline.

Dominant sediment types were derived from the soil data. The Harmonised Africa Soil Map (Dewitte et al. 2013) was used to derive the dominant sediment types. Properties of the representative soils along the segment of the shorelines were collated and particle size fraction data were examined to derive the most dominant particle size.

Wave exposure was computed using the Wave fetch model (Burrows, Harvey & Robb 2008). This is a first-order estimate of wave exposure. The model is based on the understanding that the larger the fetch area for each shore unit the greater the wave exposure. The model requires identification and classification of the shoreline, water and land areas, from which fetch values for each of the 16 angular sectors were calculated. The sum of the fetch values for each of the shoreline grid cells was used to represent the wave exposure.

### Method

The study used a combination of NOAA guidelines (NOAA 2016) and the standard set by Oil Producing Trade Section as cited in Adeofun and Oyedepo (2011) for the shoreline. In order to construct the GIS-based ES, three stages of operation were followed: identification and collation of required data for classification, definition of the knowledge-based rules and GIS-based modelling of the rules.

**FIGURE 2:**
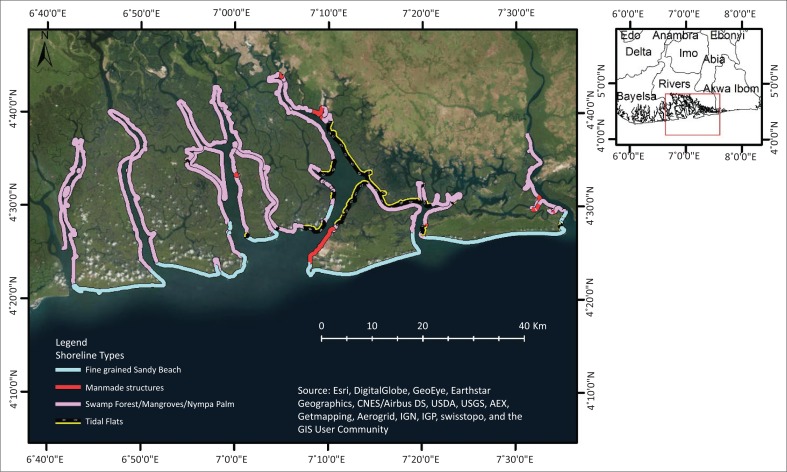
Broad classification of shoreline types from satellite imagery.

Using clearly identifiable characteristics of units along the shoreline, shore types were identified and partitioned (Figure 2). Prior to the computation of the slope, sinks and holes within the Digital Elevation Model (DEM) were filled within ArcGIS (ESRI 2015). The slope was computed in degrees within the ArcGIS and classified into four different classes – low slope (< 5), moderate slope (5–8), steep slope (8–15) and very steep slope (> 15).

The soil particle fraction with the highest proportion was extracted to represent the dominant sediment types for the soil types found along the shoreline. From this, sediment types could be defined as sand, silt or clay. In addition, areas with man-made structures along the shoreline were classified as having solid aggregate sediment type.

Wave Exposure as represented by the sum of fetch length was grouped into three classes (Table 1): low, moderate and high, based on the natural breaks classification of Jenks (1967). With this, the three wave exposure classes are low (2.00 ≥ *x* ≤ 124.01), moderate (240.65 ≤ *x* > 124.10) and high (> 240.65). Slope, exposure and dominant sediment types were all initially in raster format while the shoreline types were stored in vector format. The shoreline type was converted to point data whereby each segment is subdivided at 1 m intervals. The point data file was subsequently used to extract values from the raster-based data sets (Slope, Exposure and Dominant Sediment types).

**TABLE 1:** Distribution and length of wave exposure classes along the shoreline.

Wave exposure class	Length (km)
Low	11.30
Moderate	40.70
High	48.00

**FIGURE 3:**
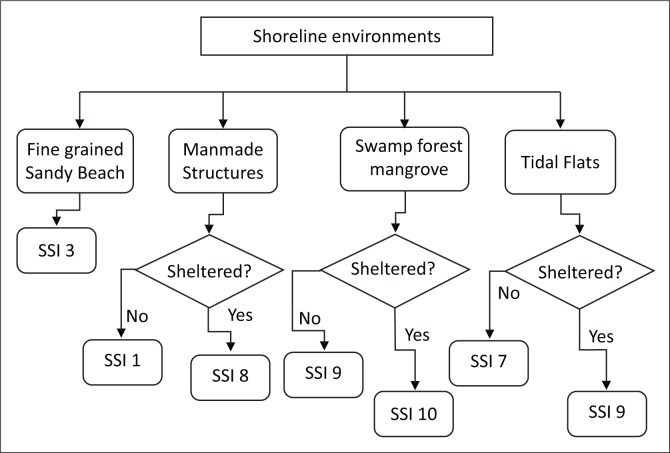
Decision tree for shoreline sensitivity ranking.

The second stage of the operation involved the definition of the knowledge-based rules. The rules were adapted and modified based on the Oil Producers Trade Section (OPTS) classification and the NOAA guidelines. The shoreline sensitivity index (SSI) is organised into 10 classes, with the highest values indicating the highest sensitivity to oil spill. Based on this, each shoreline unit was assigned SSI based on the sensitivity index with the closest match to the guidelines (OPTS and NOAA). A decision tree was used to define guiding rules for assigning SSI values, based on the combination of the attributes collated. The decision tree is represented in Figure 3 and the rule definition started from shore types and ended with the wave exposure.

Computed fetch data were used in the confirmation of the wave exposure of the shoreline; thus, shoreline segments with low relative wave exposure were considered as sheltered and all others were considered as exposed (i.e. medium to high wave exposure).

The data sets were stored and manipulated within ArcGIS, and the ranking illustrated in the query language using logical operators. Each segment (rule) of the decision tree was programmed to ascertain the conditions required for a positive identification and subsequent assignment of SSI value. Essentially, the knowledge-based rules were implemented by an ES using a conditional algorithm in association with GIS-stored spatial data. The ES was modelled within SPSS (IBM Corp. 2015).

## Results and discussion

The collated data set and the subsequent processing result in a 728 km shoreline of the study area. The distribution of the shoreline types gave a clear indication that 70% of the entire shoreline can be classified as having swamp forest/mangroves/nympa palm, which represents a total of 509 km (Table 2). Furthermore, a total of 129 km and 70 km were identified as fine grained sandy beach and tidal flats, respectively, while shore lined with man-made structures was found on around 3% of the total shoreline length. Recovery time is greatly impacted by the type of shoreline (ITOPF n.d.). Mangroves have an indicative recovery period of over 10 years, and sand beaches have a recovery period of between 1 and 2 years after oiling. About 70% of the shoreline examined was classified as swamp forest/mangroves/nympa palm. With this, there is a need for considerable effort and attention to forestall oil spills as well as initiation of prompt action in the case of oil spill disaster across the region.

**TABLE 2:** Distribution and length of shoreline types across study area.

Shore types	Length (km)
Fine grained sandy beach	128.83
Man-made structures	22.97
Swamp forest/mangroves/Nympa Palm	509.12
Tidal flats	66.93

Dominant sediment types were found to be mainly silt and clay (Table 3), while a small proportion (3.3%) of the shoreline having sediment types broadly classified as solid aggregates (wood, concrete, sand bags, sheet pile, boulders, cobbles – essentially made up of large aggregate materials).

**TABLE 3:** Distribution and length of sediment types across the shoreline.

Sediment types	Length (km)
Clay	168.86
Silt	534.97
Solid aggregates	24.02

Surface particle sediments serve as potential areas of adsorption by different cations and anions. Therefore, the more the surfaces available for these to adhere to, the higher the chances that there would be more of these in the sediments. Sediments are ultimately sinks of metals and particulate matter that are present in the aqueous phase. Fine particle sediments, especially clay and silt, are generally rich in organic content and often have higher cation exchange capacities and are able to trap metal ion, while sandy type sediments, being organically poor, have little ability to retain metal ions (Liao, Selim & Delaune 2009). The larger surface area of silt and clay makes them more chemically active than sand. Clay dominated sediments will ultimately have lower permeability which could reduce oil penetration. Furthermore, the depth of penetration is lower for silt and clay sediment because of the size of the particle, while the depth of penetration is high for sandy sediments. In essence, coarse sediments are likely to allow greater penetration than finer materials. Finer materials could also limit the effects of wind and wave action in carrying out natural clean-up as the materials are tightly packed compared with coarse materials. With most of the shoreline having silt and clay sediments, this portends serious consequences for long-term retention of materials from oiling condition, thereby making the clean-up much more difficult.

Inclination of the shoreline shows that about 476 km of the shoreline belongs to the low slope class, while about 135 km, 100 km and 17 km belong to the moderate, steep and very steep slope classes, respectively (Table 4). Inclination of the shoreline influences reflection and breaking of waves, consequently affecting the width of the intertidal streak. In essence, steep areas of the shoreline have enhanced natural clean-up potential compared to areas with lower inclination which often allows oil to stand for a long time (as the wave energy is often dissipated further, very little reaches the shoreline). From the result, it is evident that most segments of the shoreline have very little natural clean-up potential in relation to the slope, as 65% of the shoreline segments have low slope. Thus, there is a tendency for oil to stand for a long period because of very low natural clean-up by wave action as predicated by the slope.

**TABLE 4:** Distribution and length of different slope classes across the shoreline.

Slope class	Length (km)
Low	476.01
Moderate	134.65
Steep	99.71
Very steep	17.47

In the case of wave exposure, the majority of the shoreline digitised falls within the moderate and high wave exposure classes – 40.7% and 48%, respectively. Exposure is important to harness the natural ability of waves to carry out natural clean-up as well as habitat establishment and development (high wave exposure influences population and types of organisms found in such regions). The approach adopted in this study considered all the types of environment (Marine, Estuarine, Lacustrine and Riverine) based on the sum of fetch length. This provides a uniform classification frame for comparison of exposure across the shoreline. From the result, about 89% of the shoreline is exposed, with the indication that in the case of oil spills natural wave action could aid the clean-up process. However, the inclination, sediment and shoreline types would significantly impede such action. Essentially, the high wave exposure also has an implication for the spread of the oil spilled, subject to the viscosity of the oil, wind speed, water temperature, current as well as tidal stream (UNEP 2016).

**FIGURE 4:**
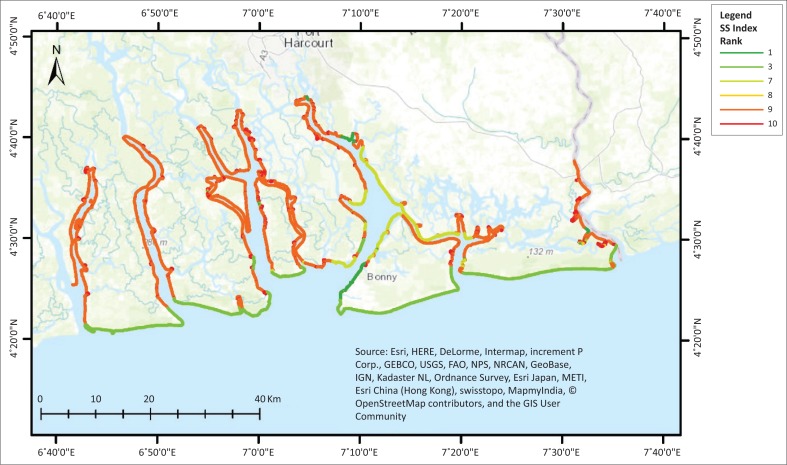
Shoreline Sensitivity index to oil spill for the study area.

The distribution of the SSI based on the decision tree (Figure 2) shows that about 61% of the entire length of the shoreline in the study area have a sensitivity rank of 9 (Figure 4), which amounts to about 446 km of very high sensitivity shoreline (Table 5). The number of segments across this dominant rank is also high compared with other ranks. However, Rank 10 was found across just about 66 km of the entire shoreline, and it is very fragmented having 652 segments over this length. These segments represent the extremely sensitive units within the area. The high level of fragmentation for this rank is directly related to the interspersion of many man-made structures across the shoreline in this region. These structures include oil and gas infrastructure, settlements, et cetera.

**TABLE 5:** Proportion of shoreline sensitivity of ranking in the study area.

Rank	Length (km)	Number of segments
1	18.92	50
3	141.20	18
7	51.68	76
8	4.37	48
9	446.17	765
10	65.51	652

Rank 1 accounts for just over 2% of the entire length of the shoreline, whereas Rank 3 was found across 19% of the entire shoreline. Rank 8 has the smallest coverage with just over 4 km of the shoreline having characteristics fitting this rank.

The sensitivity ranking gave an indication that a significant proportion (78%) of the shoreline studied is highly sensitive to oiling (Ranks 7, 8, 9 and 10); thus, this makes it very important that vigilance and adequate monitoring should be taken seriously. This also raised serious concern about the resources required in case of oiling across these segments of the shoreline. A spatial query of the Oil Spill Monitor Database could reveal the number of incidences across these highly sensitive shorelines. This could essentially offer a clue to where highly vulnerable segments are located, as well as offer an insight into why it happens in these places and the potential solutions to limiting the number of incidences.

## Conclusion

The results gave a clear indication that the alluvial plains of Rivers State have a complex mixture of sensitive environments to oil spill. The GIS-based ES with classification rules for shoreline sensitivity provided a rapid and flexible framework for automatic ranking of shoreline sensitivity to oiling based on known standards. This SSI, when incorporated with data on biological and human resources, would provide a complete picture of the environmental sensitivity to oil spills across different environments in this vulnerable region.

As the data set is stored within a GIS environment, there is a clear opportunity to update the GIS database, thus making it possible to access up-to-date information relevant for emergency operation and response. With the ES in place, new data obtained from remote sensing and fieldworks can be quickly added to the database, thereby allowing for rapid assessment of ongoing emergency situations as well as allowing for taking proactive measures to forestall potential hazardous events.

The GIS-based ES for shoreline sensitivity presents an opportunity for prioritisation of clean-up operations, intervention measures, planning as well as supporting evidence-based decisions on clean-up and containment procedures applicable for affected environments. The study adapted the combination of the Nigerian and the NOAA standards for the ranking of shoreline sensitivity. It is expected that the application of this approach within a spatial database supported by an ES would be extended to cover all of the oil producing regions of the country.

Results from this study gave an indication of the potential of the approach in supporting response teams and regulatory agencies. Essentially, this is an endeavour of supporting decision-making for clean-up activities, damage evaluation as well as supporting environmental advocacy in the oil producing region.
